# Genetic structure of Mataco-Guaycurú speakers from Argentina and the extent of their genetic admixture with neighbouring urban populations

**DOI:** 10.1038/s41598-019-54146-6

**Published:** 2019-11-26

**Authors:** Andrea Sala, Mariela Caputo, Daniel Corach

**Affiliations:** 1 Universidad de Buenos Aires, Facultad de Farmacia y Bioquímica, Departamento de Microbiología, Inmunología, Biotecnología y Genética, Cátedra de Genética Forense y Servicio de Huellas Digitales Genéticas, Buenos Aires, Argentina; 2 National Research Council-CONICET, Buenos Aires, Argentina

**Keywords:** Genetic variation, Structural variation

## Abstract

Argentina hosts more than 30 Native American groups, who are widely distributed throughout the country. Mataco-Guaycurú speakers settled in the ecoregion of Gran Chaco and represent 26.7% of the extant aboriginal population of the country. To further investigate the genetic attributes of these speakers, we focused our attention on four aboriginal groups, namely, Wichí, Toba, Pilagá and Mocoví, belonging to the Mataco-Guaycurú linguistic group. Our main goal was to evaluate the interrelationships among the groups and the relationships of these groups with admixed urban populations and to assess correspondences between molecular analysis and historical information. A total of 890 samples (282 Native Americans and 608 inhabitants of admixed urban areas) were analysed. Genetic information was gathered from 15 autosomal STRs, 17 Y-STRs, entire mtDNA control region sequences, 24 AIM-SNPs and 46 AIM-DIPs. Native American signatures were detected in 97.9% of mtDNA lineages, 89.1% of Y-haplotypes and 90.3% to 96.9% of autosomal markers. Wichí exhibited the genetic composition with the largest Native American contribution among the groups and a weak signal of gene flow. This work provides extended genetic information of potential interest in the fields of molecular anthropology and forensic genetics.

## Introduction

The demographic history of Argentina is the result of multiple migration events. Initially, Native American settlers, migrating southward, arrived at the region presently occupied by Argentina over 12,000 years ago. During this period, the migrating tribal groups differentiated by developing unique cultures and languages. The indigenous population settled in this region is estimated to have consisted of 300,000–500,000 people at the time when the first large group of European conquerors arrived. The overall distribution was uneven, with a higher population density in the west, flanking the Andes, and a lower density in the “pampas” and Patagonian regions^1^. Subsequently, admixture between Native Americans and European conquerors began, followed by Native American admixture with the colonists, resulting in new demographic categories including mestizos (admixed between Native Americans and Europeans), criollos (European descendants born in the Americas), mulatos (admixed between Europeans and Africans, forcibly introduced as slaves since the 17^th^ century), sambos (admixed between Native Americans and Africans) and many others resulting from the admixture between these new categories. Progressively, a new dominant population (consisting mainly of criollos) displaced the autochthonous populations (indigenous inhabitants) to outlying areas. The conquest followed by colonial expansion displaced aboriginals and reduced the geographical areas occupied by them, who consequently experienced a strong reduction in number due to persecution, death and subjugation.

The Gran Chaco (“Chaco” means “hunting territory” in the *Quechua* language) is a South American eco-geographical region that includes North-Central Argentina, Bolivia, Paraguay and southern Brazil. This extended area is located between latitudes 16°50′S and 33°50′S and between longitudes 67°50′W and 57°50′W, covering an area of approximately 1,391,000 km^2^. Aboriginal populations inhabiting the Argentinean part of the Chaco region were the last to submit to the power of the Spaniards and then to the national government.

When the Spaniards arrived in Argentinean Chaco in the 16^th^ century, there were three cultural groups: the so-called “typical chaquenses” (Mataco-Mataguayo and Guaycurú); the group in the jungle, immigrating from Amazonas (Tupí-Guaraní and Arawak); and the group in the mountains or Andean region (Lule-Vilela)^2,3^.

The tribes of Guaycurú speakers, including Toba, Pilagá, Mocoví and Abipón (extinct), became equestrian after the introduction of horses by the Europeans between the 16^th^ and 17^th^ centuries. This novelty, adopted primarily by Toba and Mocoví, facilitated their expansion to the south. Mataco (Wichí) did not incorporate the horse and remained pedestrian in the northwestern region of the Argentinean Gran Chaco.

The Mataco-Guaycurú linguistic branch included twelve languages spoken in Argentina, Bolivia, Brazil and Paraguay. There are no clear relationships between Mataco-Guaycurú languages and other aboriginal languages; some authors related them to Macro-Ge^4^, while others related them to Macro-Panoan^5^.

Currently, according to the last Argentinean Supplementary Survey of Indigenous Peoples^6^, there are 40,036 Wichí (90.3% of the Wichí who inhabit the country) inhabiting Chaco, Formosa and Salta Provinces; 69,452 individuals of Toba ethnicity (68.5%) inhabiting Chaco, Formosa and Santa Fe Provinces; 4,465 Pilagá (88.4%) living in Formosa Province and 15,837 Mocoví (76.7%) living in Chaco and Santa Fe Provinces. In total, these ethnicities constitute approximately 26% of the extant Native American populations inhabiting Argentina.

In this work, we analysed the genetic relationships between different groups of Mataco-Guaycurú-speaking tribesmen. We used a large set of polymorphic genetic markers transmitted either bi- or uniparentally. The marker set included autosomal short tandem repeats (STRs), Y-chromosome STRs, the entire control region of the mitochondrial DNA (mtDNA) D-loop sequence and ancestry-informative markers (AIMs) that included deletion-insertion polymorphisms (AIM-DIPs) and single nucleotide polymorphisms (AIM-SNPs), which were used to obtain the genetic landscape of these tribal groups. To evaluate the strength of the genetic relationships between isolated tribal groups and admixed urban populations, a sample set from Formosa, Chaco, Santa Fe Provinces and the cosmopolitan population of Buenos Aires Province were also analysed. We assessed the extent of gene flow between the populations and the three major parental lineages, namely, the Sub-Saharan African, European and Native American lineages, based on the Centre d’Etude du Polymorphisme Humain (CEPH) Panel datasets.

Previous studies focusing on tribal groups inhabiting Gran Chaco analysed serological polymorphisms^7^, HLA markers^8^, mitochondrial Native American haplogroup (hg) frequencies by PCR/RFLP assays^9^, mtDNA hypervariable region I sequences^10^, a set of autosomal STRs^11^ and Y-STRs^12^. Demarchi and García Ministro^13^ summarized previous analyses^9–12^ and concluded that the Argentine population of Gran Chaco was genetically homogeneous, with intense gene flow between the Mataco and Guaycurú groups.

Aiming to broaden the scope of available genetic data, we expanded the analysed groups, the number and type of polymorphic genetic markers used and the extent of mtDNA sequences. The data obtained allowed us to analyse genetic structure and gene flow by STRs and AIMs and provided novel information concerning Y-chromosomal and mtDNA haplotypes. This work provides genetic information that can be used to better characterize the descendants of Native Americans. The large dataset provided here significantly expands existing genetic databases about the region and might be of interest to molecular anthropologists and human population and forensic geneticists.

## Results

### Admixture analysis

Aiming to investigate the genetic composition of Mataco-Guaycurú speakers as a consequence of internal migrations and after contact with European descendants, we compared genetic information across 15 autosomal STRs, 46 AIM-DIPs and 24 AIM-SNPs to estimate the degree of genetic admixture.

**Figure 1:**
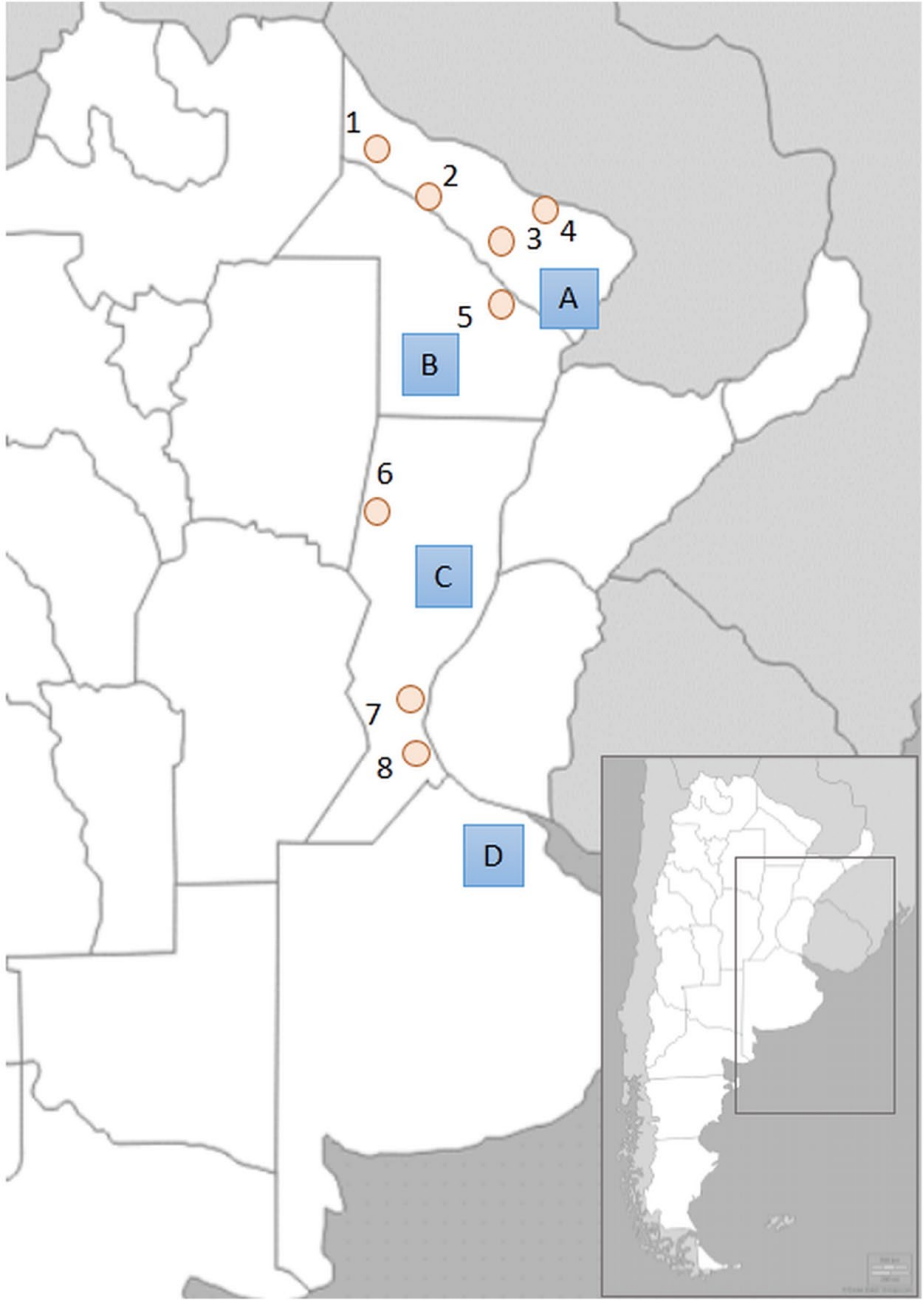
Geographic locations of sampling sites, indicating isolated aboriginal groups (circles) and urban populations (squares). Native American samples: 1-Wichí (Formosa), 2/3-Pilagá (Las Lomitas and Colonia Ibarreta, Formosa), 4-Toba (Laguna Blanca, Formosa), 5-Toba (San Martin, Chaco), 6-Mocoví (Tostado, Santa Fe), 7/8-Toba (Santa Fe and Rosario, Santa Fe). Urban samples: A-Formosa Province, B-Chaco Province, C-Santa Fe Province, D-Buenos Aires Province.

Fifteen autosomal STRs were typed in 70 Pilagá, 54 Wichí, 121 Toba and 37 Mocoví belonging to the Mataco-Guaycurú-speaking group (see Fig. 1 and Materials and methods for details on the samples). Supplementary Material 1-a shows the frequency distributions across loci, molecular indices and Hardy-Weinberg equilibrium (HWE) results as well as a summary of the autosomal STR analysis results.

We included a sample set of reference populations from the CEPH panel (Sub-Saharan African, CEPH-AFR; European, CEPH-EU and Native American, CEPH-NA) and admixed populations from the provinces inhabited by the Native American sample donors: Formosa, Chaco and Santa Fe. A sample from Buenos Aires Province was also included since this region contains the most cosmopolitan population and experienced the highest influx of internal immigration.

**Figure 2:**
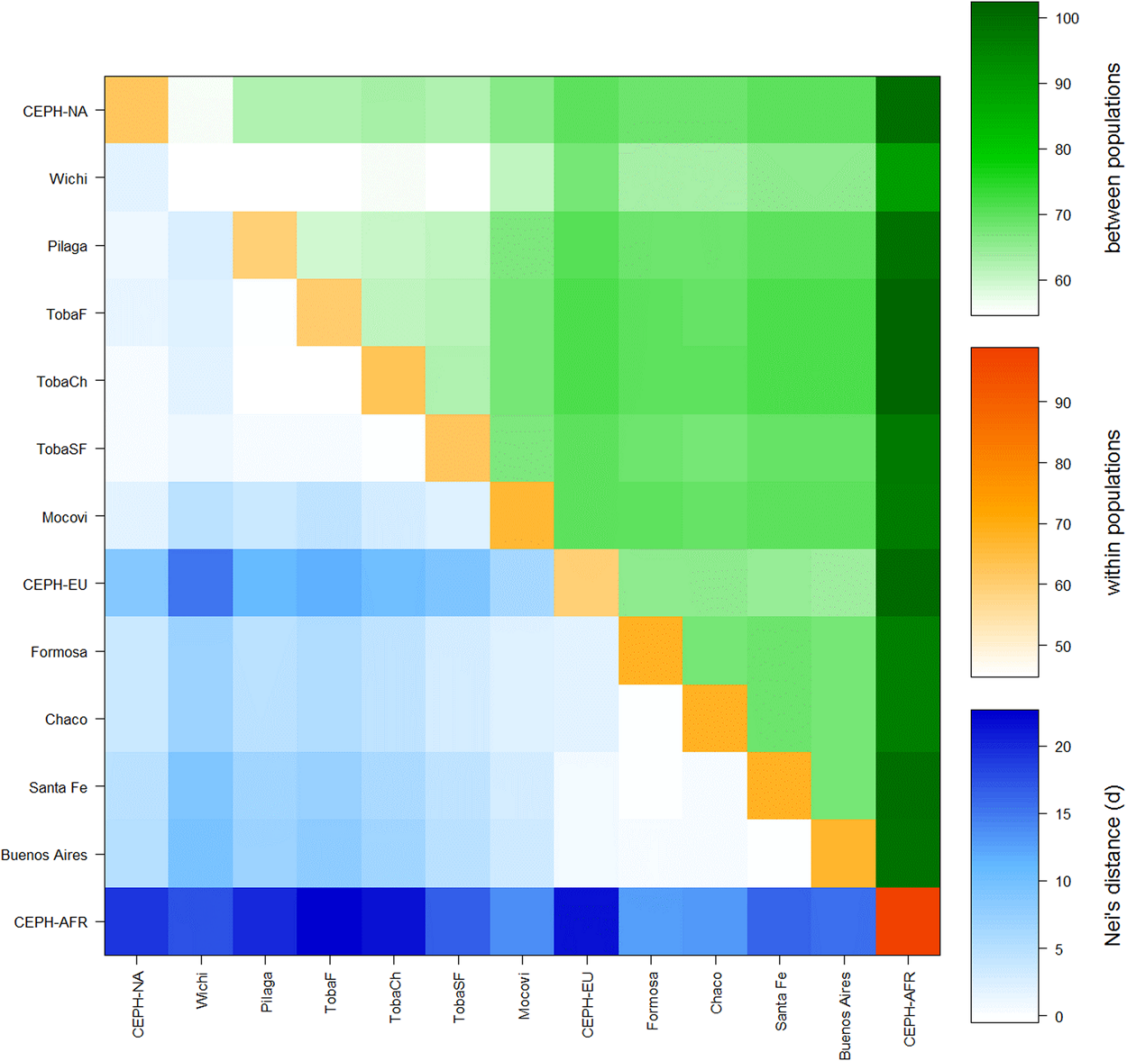
(**a**) Nei’s genetic distance, lower left triangle, coloured in blue; (**b**) the average number of pairwise differences within each population, diagonal, coloured in orange; and (**c**) between-population differentiation, upper right triangle, coloured in green. Codes: CEPH-AFR, CEPH-EU and CEPH-NA stand for the Sub-Saharan African, European and Native American CEPH parental populations, respectively; Formosa, Chaco, Santa Fe and Buenos Aires correspond to admixed samples from urban areas; and Wichí, Pilagá, Toba and Mocoví are Mataco-Guaycurú-speaking groups (Table 1).

Figure 2 summarizes Nei’s genetic distance and the average number of pairwise differences within each population and between them. The Wichí group exhibited the smallest average number of within-population pairwise differences (indicating that this group was the most homogenous, in agreement with this group having the lowest observed heterozygosity) and the lowest between-population differentiation among the Native American groups.

**Figure 3:**
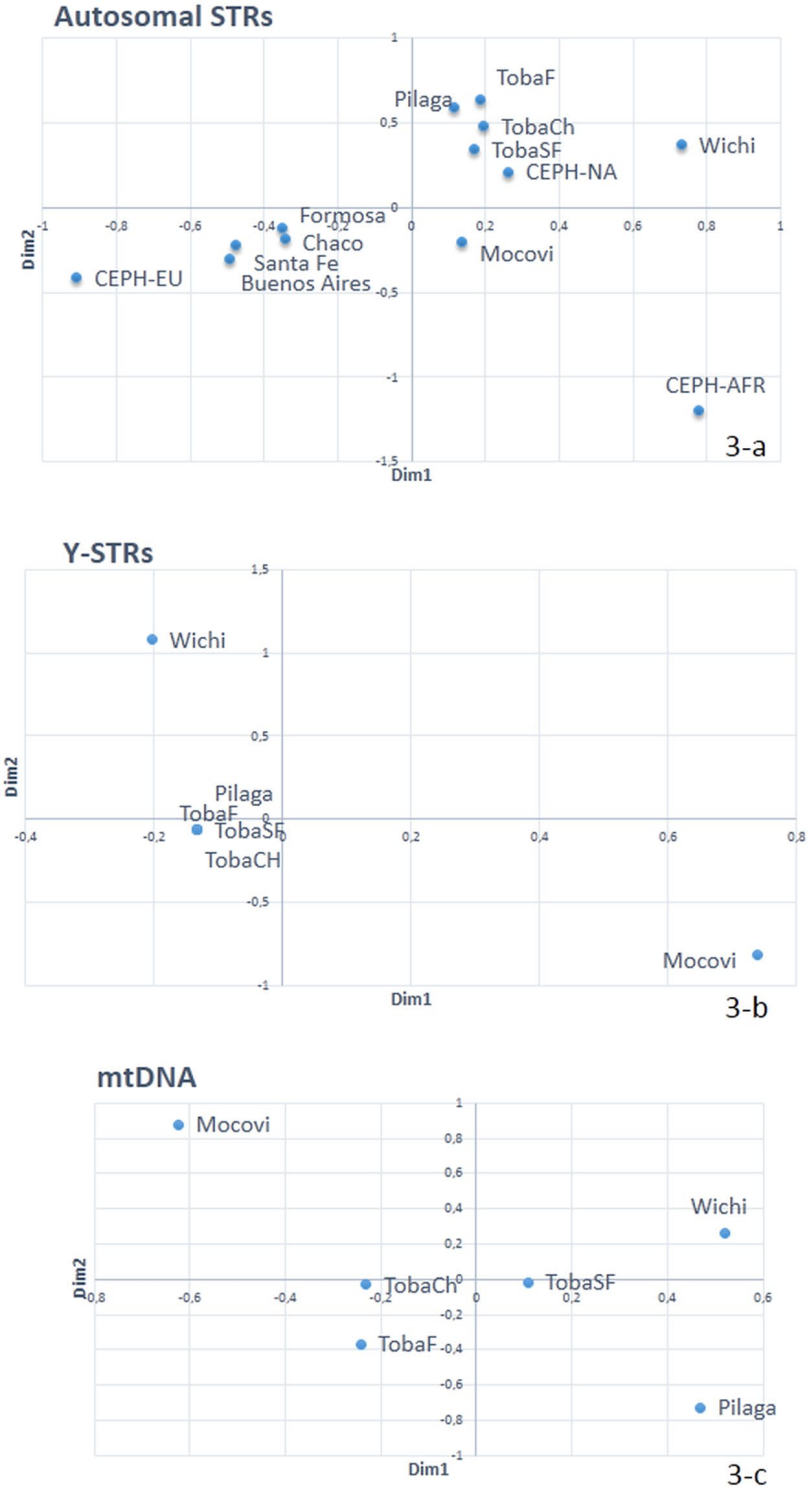
(**a**) MDS plot depicting the genetic distances based on autosomal STRs between Mataco-Guaycurú groups, Argentinean urban populations and reference groups from the CEPH panel (Kruskal stress = 0.043); (**b**) MDS plot of Slatkin’s linearized genetic distances based on Y-STR haplotypes in the Q1a3a hg (Kruskal stress = 5.541E-5); and (**c**) MDS plot of genetic distances based on mtDNA control region sequencing (Kruskal stress = 1.173E-4).

Slatkin’s linearized genetic distances, depicted in the multi-dimensional scaling (MDS) plot shown in Fig. 3a, revealed two clusters, one including Pilagá, Toba and CEPH-NA and another including urban Argentinean populations, closer to CEPH-EU. The Mocoví group was located between the mentioned clusters. The Wichí group exhibited the highest genetic distance from the rest of the Native American groups and the highest genetic distance from the urban and CEPH-EU samples; CEPH-AFR was isolated in the graph. The genetic distances of Mocoví and Wichí were statistically significant with respect to the remaining populations analysed.

AMOVA taking into account the urban population vs Toba (pooled into one group) vs the rest of the Mataco-Guaycurú groups revealed that 6.6% of the variation was among groups (p = 0.00684 +/− 0.00231) and no significant variation among populations within groups (0.11339 +/− 0.00819) (Supplementary Material 1-a).

**Figure 4:**
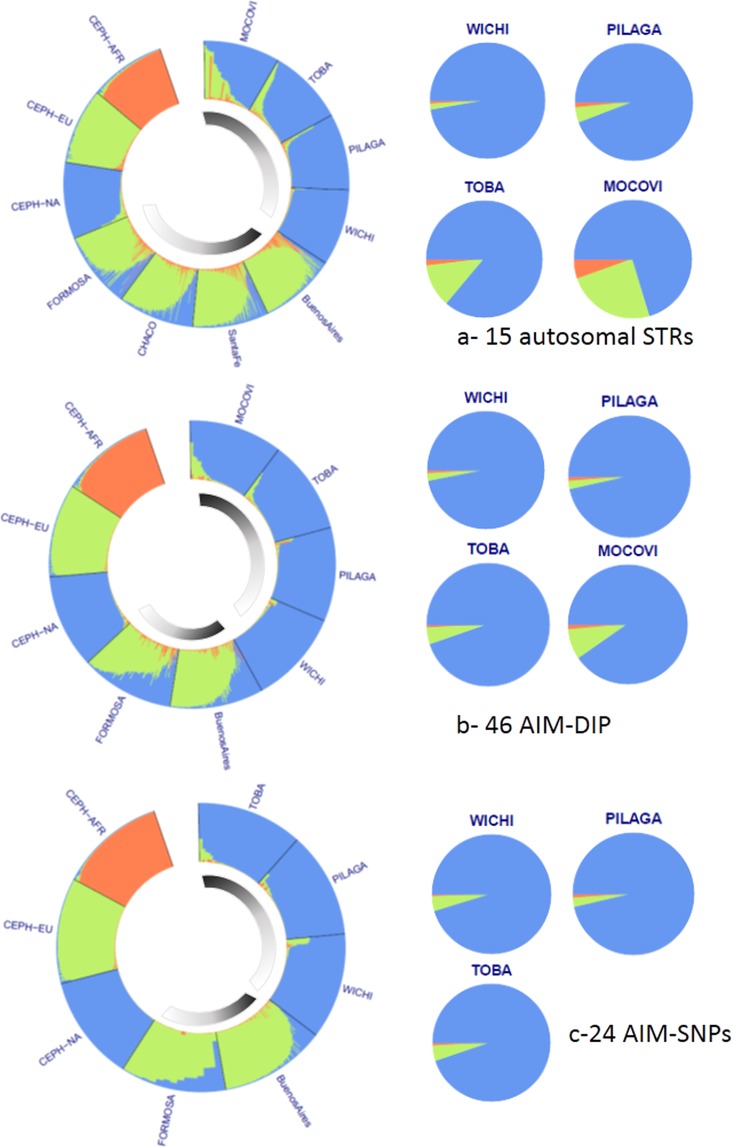
Graphical representation of admixture analysis by STRUCTURE software based on autosomal STRs (**a**), AIM-DIP (**b**) and AIM-SNPs (**c**) (see data in Supplementary Material 2-b). Colour codes: orange represents the African component; green, the European component, and blue, the Native American component. The internal semi-circular bars indicate a gradient from white to black in the north-to-south direction of the urban and Native American populations included in the analysis. Toba from Formosa, Chaco and Santa Fe are presented as the unified group “Toba”.

Genetic structure was assessed with STRUCTURE software using 15 autosomal STRs routinely used for forensic identification purposes. Table 1 summarizes the results, including those for parental populations from the CEPH panel. Although this panel of autosomal microsatellites underestimated the aboriginal component, there was a correlation between this result and those obtained with AIM-DIPs (analysed in 161 Mataco-Guaycurú samples) and AIM-SNPs (analysed in 103 Mataco-Guaycurú samples) (Supplementary Material 1-b). The AIM-DIP results provided a clear overview of the genetic structure of the populations under study. Wichí exhibited the largest Native American contribution, and Mocoví, the smallest. The Native American component decreased from north to south, i.e., from Wichí to Mocoví. No significant differences in genetic composition were observed among Toba from Formosa, Chaco and Santa Fe. The AIM-SNPs showed a good correlation with data obtained from AIM-DIPs in Wichí, Pilagá and Toba (Fig. 4 and Supplementary Material 1-b).

**Table 1:** Admixture extent in Mataco-Guaycurú groups analysed by means of 15 autosomal STRs.

Population	Ancestry component		
	African	European	Native American
CEPH-AFR	**0.9569**	0.0264	0.0167
CEPH-EU	0.0291	**0.9543**	0.0166
CEPH-NA	0.0117	0.0528	**0.9355**
WICHÍ	0.0100	0.0166	0.9734
PILAGÁ	0.0161	0.0424	0.9415
TOBA	0.0188	0.1197	0.8615
MOCOVÍ	0.0546	0.2424	0.7030
FORMOSA	0.0648	0.7198	0.2154
CHACO	0.0513	0.7052	0.2435
SANTA FE	0.0656	0.7779	0.1565
BUENOS AIRES	0.0827	0.8236	0.0937

Parental populations from the CEPH panel: African (AFR), European (EU) and Native American (NA).

**Figure 5:**
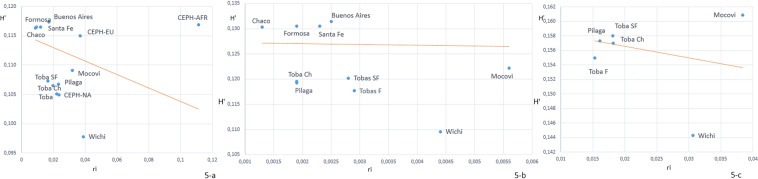
Plot of the proportion of heterozygotes (H′) in each population expected under HWE against the distance from the centroid (ri). The line represents the expected relationship predicted by the model of Harpending and Ward (1982). (**a**) Complete dataset including CEPH panels, urban populations and Mataco-Guaycurú-speaking groups; (**b**) urban samples and aboriginal groups; and (**c**) Mataco-Guaycurú-speaking groups.

Analyses of allele frequencies by the centroid method independently confirmed the results described above. Figure 5 shows the amount of gene flow experienced by a population examined by the centroid method^14^. The regression line represents the expected heterozygosity; populations plotted above the line would have received higher-than-average gene flow, while those positioned under the regression line would have been less impacted by gene flow and, therefore, would have evolved in a relatively isolated manner. The positions of the populations relative to the regression line vary widely in the panels in Fig. 5(a–c) because of a reduction in the number of groups included and the number of alleles in each dataset. As expected, Wichí was plotted far below the regression line, indicating that the gene flow experienced was substantially lower than the average computed for the whole set of populations included in the analysis. In contrast, Mocoví was located above the regression line, indicating that this group may have been exposed to more gene flow.

### Analysis of Y-STRs

Seventeen Y-STRs and the SNP M3-Q3 were analysed in 210 male samples. Supplementary Material 2 summarizes the haplotype distribution across populations, the molecular diversity indices, the haplotypes shared by groups and a comparative analysis with previously published reports^12^.

A microvariant at locus DYS385b (allele 16.1) was observed in three haplotypes in Pilagá and five haplotypes in Wichí. One of these haplotypes was the most frequent haplotype in Wichí (29.3%) and in the global Mataco-Guaycurú sample. Two of six haplotypes were previously described in the Toba group from northern Chaco Province by Toscanini *et al*.^15^ (Supplementary material 2).

**Figure 6:**
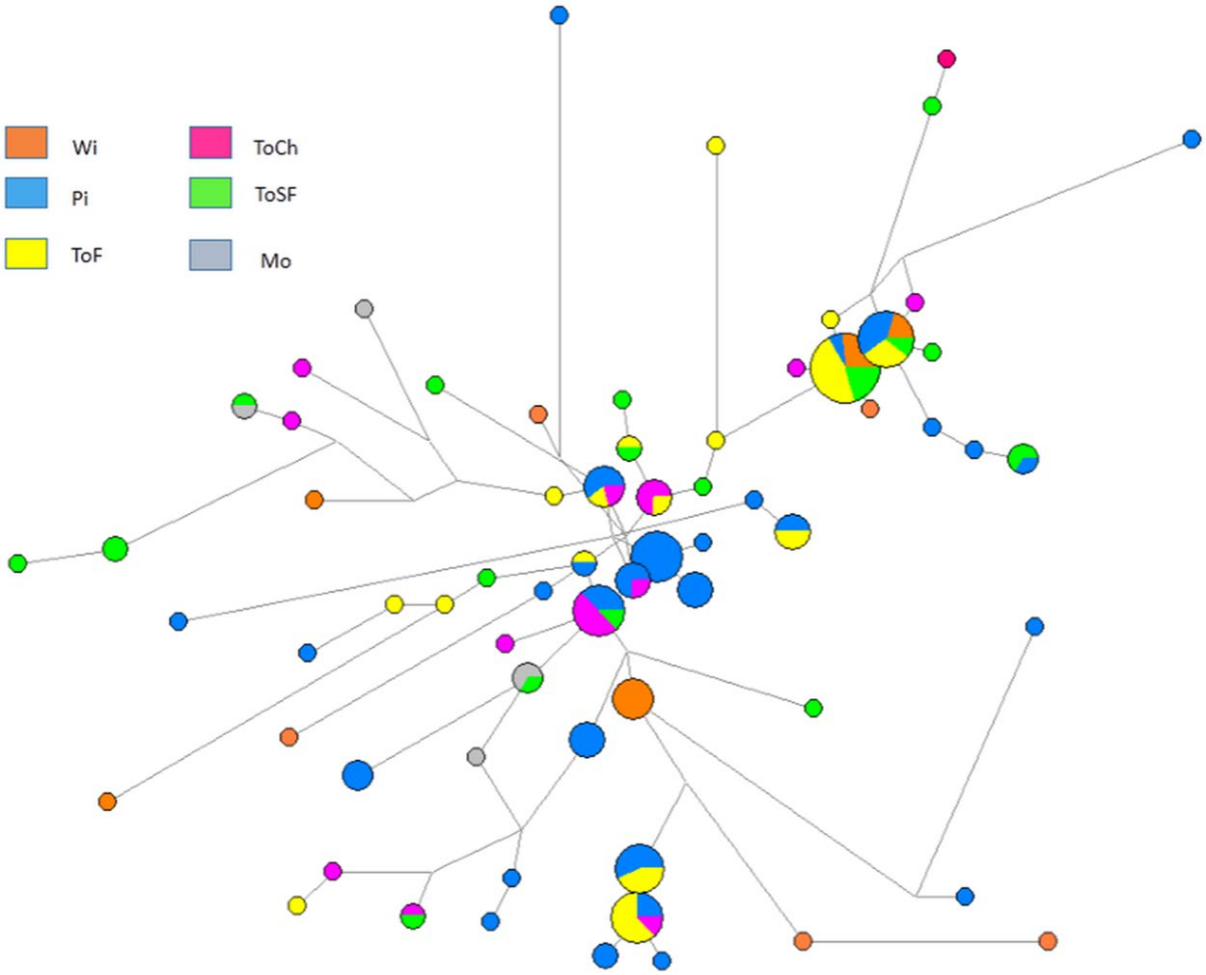
MJ network connecting Y-STR haplotypes (Q1a3a hg). Stars indicate possible founder haplotypes (DYS385b/16.1 haplotypes are excluded in this representation).

Figure 6 shows a median-joining (MJ) network of Y-STR haplotypes in Mataco-Guaycurú speakers. The network includes several star-like configurations that might indicate recent expansions of founder haplotypes. The majority of the shared Y-haplotypes were shared by Wichí, Toba and Pilagá from Formosa Province.

An MDS plot of the Q1a3a Y-haplotypes is presented in Fig. 3b. Pilagá clustered with the Toba groups. The genetic distances between Wichí and the other groups were statistically significant. In addition, statistically significant distances were observed between Mocoví and TobaF and between Mocoví and TobaCh (see Supplementary Material 2).

Only 23 of 210 (10.9%) Y-haplotypes were non-Amerindian. The number of non-Amerindian Y-hgs increased from north (Pilagá: 4/72; 5.5%) to south (Mocoví: 10/17; 58.8%).

### mtDNA analysis

A total of 245 haplotypes of the entire mtDNA D-loop region are summarized in Supplementary Material 3 (haplotype dataset, diversity indices, haplotypes shared by groups and the comparative study). Using the hg assignment tool EMPOP, we determined that 97.9% of the haplotypes belonged to Native American hgs. Only five samples exhibited non-Native American signatures.

**Figure 7:**
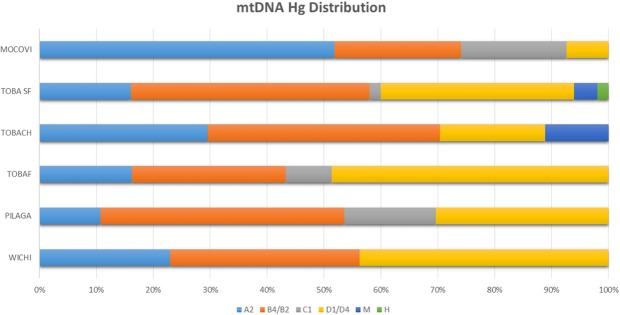
Entire mitochondrial D-loop sequence-based hg frequency distributions for Mataco-Guaycurú speakers from Argentina.

Figure 7 shows hg frequency distributions across groups. Hg B2 is well represented in all groups; hg A2 was the most frequent in Mocoví, while hg C1 was absent in Wichí and Toba from Chaco. Hgs D1 and D4j were detected in different proportions, with very low frequencies in Mocoví.

Genetic distances based on mitochondrial control region sequencing data were statistically significant in most cases, except for those of Toba-Ch with Toba-F and Toba-SF. The highest genetic distances were observed between Mocoví and all other groups (Supplementary Material 3 and Fig. 3c).

**Figure 8:**
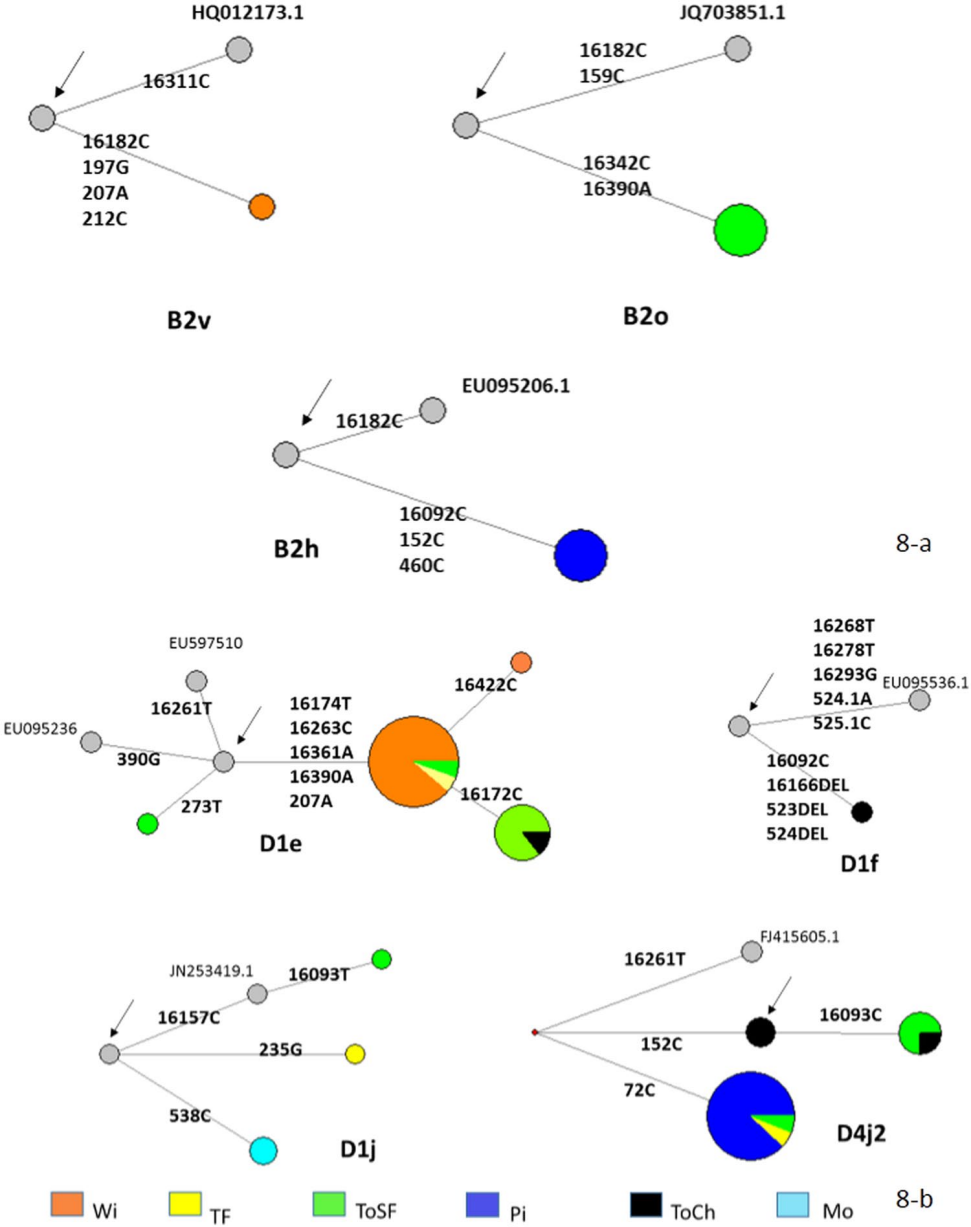
MJ network of hg B2 (**a**) and hgs D1 and D4 (**b**), connecting Mataco-Guaycurú haplotypes with nodal (indicated with an arrow) and PhyloTree-associated sequences (accession numbers are also indicated). The analysis was restricted to D-loop sequences, disregarding insertions at position 309. The deletion at 16,166 was weighted three times over transitions. Private mutations are indicated in bold. Colour codes are included in the figure.

Figure 8 depicts the networks between some hgs, including D1j (N = 4), D1e (N = 27), D4j2 (N = 23), D1f (N = 1), B2h (N = 4), B2v (N = 1) and B2o (N = 4). Mataco-Guaycurú haplotypes were related to nodal sequences as well as sequences from the PhyloTree database (http://www.phylotree.org/). Several unique mutations in the D-loop region differentiated the Mataco-Guaycurú sequences.

Sub-hg D1j was found in four samples from Mocoví and Toba but not observed in the EMPOP database. García *et al*.^16^ studied this hg, compiling a list of D1j haplotypes belonging to Argentinian populations, mainly from central areas of the country. In this study, García proposed a local origin of D1j in the Sierras Pampeanas region of Argentina. In addition, this hg was observed in the Mapuche group (Sala and Corach^17^, N = 2/39). Notably, the haplotypes observed in the present work differed from all previously described haplotypes due to their unique mutations (T538C, T16093C and T16157C).

Sub-hg D1e was observed in 27 samples from the Toba and Wichí groups. Disregarding insertions at 309, one group of 17 samples and another of 7 samples were identical (including private mutations). In addition, three single haplotypes, two with different private mutations and one identical to the nodal haplotype, comprised the D1e set in the Mataco-Guaycurú sample. This sub-hg was observed twice by Bobillo *et al*.^18^ (in northeastern Argentina) and in the Chiriguano Native American group^19^, all with different sequences.

Sub-hg D1f was observed in only one sample of Toba from Formosa, with a deletion at position 16,166. This haplotype does not match the other D1f samples previously described by Bobillo *et al*.^18^ or García *et al*.^16^.

Sub-hg D4j2 was observed in 8 Toba samples and was the most frequent hg in Pilagá (15/56). This hg was observed in one sample from southern Argentina^18^ but has not been observed in any other aboriginal group previously investigated by our team: Mapuche and Tehuelche^17^, Chiriguano^19^ or Mbyá - Guaraní^20^.

Regarding sub-hgs belonging to the B2 branch (Fig. 8a), we found sub-hg B2h in 4 Pilagá samples, B2v in one Wichí sample and B2o in 4 Tobas samples. These sequences represent unique mutations that were not found in other Native American groups studied by our team or in the EMPOP database. The sub-hgs B2o and B2h were described previously in Argentina (B2o in 3 samples from Jujuy, northern Argentina, by Cardoso *et al*.^21^; B2h in 2 samples from Buenos Aires by Bobillo *et al*.^18^).

Other sub-hgs, including A2ag (in one Mocoví) and A2ah (in one Toba), were not found or were infrequent in other Native American groups from Argentina (A2ah was observed once in Chiriguano by Sala *et al*.^19^).

## Discussion

Genetic data emerging from this study of extant Mataco-Guaycurú-speaking groups inhabiting the ecoregion of “Gran Chaco” in Northern Argentina allowed us to gather novel information that broadens the scope of present-day knowledge about Mataco-Guaycurú speakers.

In agreement with the conclusion of Demarchi *et al*.^13^, no statistically significant differences were found between the Toba groups that inhabit different provinces. This result might reflect a homogeneous group due, in part, to the fact that the Toba people speak a common language (Qom) and to recent migration events that led to their present-day dwellings.

In clear contrast with the homogeneity of Toba, the other groups showed peculiar attributes concerning genetic structure, gene flow and uniparentally transmitted markers.

As expected, and based on historical records, the gene pool of Wichí contained the largest aboriginal component among the groups; this group was the most genetically distant from the other groups. Isolated conditions were reflected in all the polymorphic genetic markers tested and underscored by the centroid method, revealing the lowest gene flow among the aboriginal groups included herein. In addition, the smallest allele size range and number of alleles per locus for all the studied loci were observed in Wichí. The hypothesis that a recent bottleneck can still be detected in these groups is being evaluated by expanding the number of DNA markers (manuscript in preparation).

The Native American component decreased from north to south, i.e., from Wichí to Mocoví. This pattern can be explained by the higher internal migration experienced by Toba and Mocoví because of the incorporation of the horse as a tool of power and conquest during colonial times. In contrast to these groups, Wichí remained pedestrian in the northern range of their distribution, in Formosa and Salta Provinces, with reduced contact with other groups.

Currently, the natural environment is severely affected by intense farming and highly technical agricultural practices, forcing Native American descendants to migrate to urban areas to search for work opportunities. Table 2 summarizes the aboriginal composition of the groups analysed.

**Table 2:** Bi- and uniparental aboriginal compositions of Mataco-Guaycurú speakers included in this study.

Province	Ethnic group	Native American component			Demographic information
		Autosomal AIM-DIP	Y-chromosome	mtDNA	Population per ethnicity/total population per province (%)
	Wichí	96.9%	100%	100%	2.97%
Formosa	Pilagá	96.1%	94.5%	100%	0.89%
	TobaF	94.9%	91.7%	100%	2.51%
Chaco	TobaCh	94.9%	100%	88.9%	3.12%
Santa Fe	TobaSF	94.1%	77.8%	96%	0.47%
	Mocoví	90.3%	41.2%	100%	0.45%

Demarchi and Mitchel^12^ reported that 52.6% (10/19) of Pilagá sample donors carried a non-Native American-specific Y-hg. In contrast, we found that only 5.5% (4/72) exhibited the non-Amerindian signature on the Y chromosome. This difference may be due to a difference in sample sizes and/or sampling sites (see the comparative analysis in Supplementary Material 2). Such a discrepancy substantially affects the overall genetic landscape obtained as well as the reconstructed relationships of Pilagá with other ethnic groups. Although Pilagá did not undergo strong migratory movements, they had more interactions with Toba, possibly explained by their linguistic affinity, since both groups belong to the Guaycurú linguistic sub-branch, and their geographical proximity; both factors might promote a certain degree of gene flow. These patterns were reflected in the genetic distances between the Pilagá and Toba groups (p > 0.05) based on both autosomal STRs and Y-STRs.

Microvariant DYS385 16.1 was observed in six different haplotypes in Pilagá and Wichí, but we did not find it in the Toba groups, as did Toscanini^15^. Network configuration showed a possible founder haplotype carrying this variant and connecting the three ethnicities (HY1 haplotype, in Supplementary Material 2).

The statistically significant mitochondrial genetic distances between most of the analysed groups may indicate less migration of women than of men, compatible with the hypothesis of matrilocality.

Cabana *et al*.^10^ reported results of mtDNA analysis of Pilagá, Toba and Wichí ethnicities based on hypervariable region I, with N = 204. These authors found that 48/204 (23.5%) haplotypes differed, with 31% shared by different Mataco-Guaycurú-speaking groups. The additional genetic information provided herein (entire D-loop sequencing data) allowed us to increase the information about this marker, such as the haplotype diversity and haplotype discrimination power within each hg (see the comparative analysis in Supplementary Material 3). The haplotype distribution reported here is similar to that reported by Cabana, except for the absence of hg C in Wichí and in Toba from Chaco (present in Cabana’s study). These results could be explained by a difference in sample sizes or sampling locations (in Cabana’s publication, the sampled geographical location was not clear).

Recently published work on the Alto Paraná region of Paraguay^22^ reported some of the abovementioned hgs (B2b3a, 2%; B2o, 1%; B2h, 4%; D1e 4% and D1f, 1%), although the D-loop haplotype sequences were different from those of Mataco-Guaycurú reported in this study.

Based on autosomal and patrilineal compositions (55.5% of Y-haplotypes were non-Amerindian), Mocoví were the most admixed group. Furthermore, genetic distance representation via MDS plots (Fig. 3a) placed the Mocoví group between the aboriginal and urban populations. This observation was also reflected in the extent of gene flow, as shown in Fig. 5c, where only the Mocoví group was placed above the regression line. However, Native American hg signatures in maternal mtDNA were present in 100% of the analysed Mocoví, indicating clear bias in the admixture process, as reflected for most areas of the country and the continent.

Statistical information obtained from population genetics analysis is presented in the supplementary information since it might be of interest in molecular anthropology as well as for forensic purposes. Some Y-STRs and mtDNA haplotypes found in the analysed populations have been uploaded to specific databases, such as the Y-chromosome Haplotype Reference Database (YHRD) and EMPOP. Further sequencing of the full mitochondrial genome could expand knowledge of the sub-hg diversity of the aboriginal populations investigated.

This work complements previous research carried out by other groups and by our team, who have worked on the genetic characterization of different aboriginal groups inhabiting Argentina and whose main goal is to increase knowledge about the native populations that persist despite the multi-ethnic admixture process taking place in present-day Argentina.

## Materials and Methods

### Individuals

Sample donors read and signed a written informed consent statement. Research projects and consent statements were approved by the Bioethics Committee of the School of Pharmacy and Biochemistry, Buenos Aires University, Argentina (Res. 1053, Expte 744085/FFyB-UBA). The research was performed in accordance with relevant guidelines, and signed informed consent was obtained from all donors. A set of 890 individuals was analysed, including 282 Native American tribesmen who spoke Mataco-Guaycurú and inhabited three Argentinean provinces (Formosa, Chaco and Santa Fe), 608 individuals from urban populations of four provinces (the abovementioned provinces and Buenos Aires Province), and three parental populations from the CEPH panel. Table 3 provides a summary of population codes, ethnicities, geographical locations, provinces and sample sizes. Figure 1 depicts the geographic location of the sampling sites.

**Table 3:** Sample codes, ethnic group names, sampling sites, geographic locations, provinces and sample numbers analysed in the present work.

Pop. code	Ethnicity	Geographic location	Province	N*
Pilagá	Pilagá	Localities: Colonia Ibarreta, 25°13′0″S, 59°51′0″W and Las Lomitas, 24°42′26″S, 60°35′40″W	Formosa	72
Wichí	Wichí	Localities: Ingeniero Juarez, 25°10′60″S, 58°7′60″W and El Yacaré, 23°38′42″S, 62°15′01″W	Formosa	54
TobaF	Toba	Locality: Laguna Blanca, 25°07′46″S, 58°14′70″W	Formosa	38
TobaCh	Toba	Locality: General Jose de San Martin, 26°32′22″S, 59°20′32″W	Chaco	26
TobaSF	Toba	Localities: Rosario, 32°57′13″S, 60°39′84″W and Santa Fe Capital, 31°37′47″S, 60°41′59″W	Santa Fe	57
Mocoví	Mocoví	Locality: Tostado, 29°13′84″S, 61°46′10″W	Santa Fe	37
FOR	Admixed	Urban area, 26°11′5″S, 58°10′33″W	Formosa	147
CHA	Admixed	Urban area, 27°27′5″S, 58°59′12″W	Chaco	118
SFE	Admixed	Urban area, 31°38′0″S, 60°42′0″W	Santa Fe	178
BA	Admixed	Urban areas, 34°35′59″S, 58°22′55″W	Buenos Aires	165
CEPH-AFR	African	Bantu Kenya, Biaka Pygmy, Mandenka, Mbuti Pygmy, San and Yoruba		70
CEPH-EU	European	Basque, French, Italian, Sardinian, Orcadian, Russian and Tuscan		84
CEPH-NA	Native American	Colombian, Karytiana, Surui, Maya and Pima	50	

*N: Number of unrelated individuals.

### Analytical methods

#### DNA extraction

DNA was extracted from either blood samples or liquid saliva by using conventional protocols^23,24^.

#### Autosomal STRs

We typed Amelogenin and fifteen STRs: D3S1358, TH01, D21S11, D18S51, Penta E, D5S818, D13S317, D7S820, D16S539, CSF1PO, Penta D, VWA, D8S1179, TPOX, and FGA. Experimental procedures and analyses were performed according to the manufacturer’s protocol (PowerPlex 16 System kit, Promega Corp., Madison, USA).

#### Y-chromosome STRs and Y-SNPs

We analysed seventeen Y-STRs included in the AmpFlSTR YFiler Kit (Thermo-Fisher, Foster City, USA) in all the samples, except in TobaSF and Mocoví samples, which were analysed with twelve Y-STRs included in the PowerPlexY kit (Promega Corp.).

The haplotypes included in this study have been deposited in the YHRD (http//www.yhrd.org; accession numbers: YA003004, YA003005, YA003802, YA002989, YA004639 and YA004640, available in the next update- R62-).

The Y-SNP M3-C/T (Q1a3a1 hg) was analysed by real-time PCR followed by high-resolution melting analysis as previously described^25^.

#### AIM-DIP and AIM-SNP analyses

A total of 307 reference samples from the CEPH panel, 161 samples from Mataco-Guaycurú speakers (31 Wichí, 41 Pilagá, 57 Toba and 32 Mocoví) and 200 samples from the urban populations were typed with a panel of 46 AIM-DIPs. In addition, 247 reference samples, 103 Mataco-Guaycurú samples and 162 urban population samples were analysed with 24 autosomal SNPs (AIM-SNPs). A total of 46 AIM-DIPs and 24 AIM-SNPs were amplified as previously described^26,27^. The analysed samples as well as results from the structure analysis are summarized in Supplementary Material 1b. Given the homogeneous genetic structure observed in Toba from Formosa, Chaco and Santa Fe, the data were treated as a single group called Toba.

#### mtDNA D-loop sequencing

We sequenced the entire mtDNA D-loop (1,120 base pairs). Amplification, purification and electrophoresis conditions were as previously described^19^. All sequencing analyses were performed with both forward and reverse primers (with at least four to six primers for each sample). Electropherograms were edited with Sequencher v 5.3 software (Gene Codes Corporation, USA). Hgs were determined using the EMPOP v4/R11 Query Search tool (http//www.empop.org). All sequences obtained and reported herein were deposited in EMPOP under accession number EMP00667^28^.

Table 4 summarizes the number of samples analysed for each type of marker.

**Table 4:** Summary of markers analysed in each Mataco-Guaycurú group.

	Wichí	Pilagá	Toba	Mocoví
Autosomal STR	54	70	121	37
AIM-DIP	31	41	57	32
AIM-SNP	51	26	26	—
Y-STR + Y-SNP	40	72	81	17
mtDNA seq.	48	56	101	27

### Statistical analysis

Allele frequencies, HWE, gene and haplotype diversities, genetic distances and heterozygosity were determined with Arlequin v3.5.2.2^29^.

Admixture analysis was performed with STRUCTURE v2.3.4 software^30^, employing 15 autosomal STRs^31^, 24 AIM-SNPs and 46 AIM-DIPs. Genotypes of the parental populations, including the Sub-Saharan African, European and Native American populations, were obtained from published data derived from CEPH Panel samples. For STRUCTURE analysis, five iteration rounds were used. The number of parental populations (k) was set from two to five. Monte Carlo-Markov chain simulation, including a burn-in step of 10,000 iterations followed by 20,000 iterations for data gathering, was performed for each round. The number of populations assumed was initially set to the number of parental populations. An admixture model and independent allele frequencies were used. The most likely value for the number of parental populations (k = 3) was determined using the online program Structure Harvester, which enables implementation of the Evanno method^32^. Further data analysis was performed using *CLUMPP*^33^. Graphical representation was performed with Ancestry Painter software (http://www.picb.ac.cn/PGG/resource.php; ^34^).

Gene flow extent was analysed by using autosomal STR allele frequency data by means of the centroid method proposed by Harpending and Ward^14^. Calculations were performed with Geno Cline V.1.1 software (http://genocline.sourceforge.net). Three sets of samples were considered as the complete set: CEPH Panel parental samples, urban Argentinean samples and Mataco-Guaycurú speaker samples. Then, each group was sequentially removed. Regression plots reflecting the proportion of heterozygotes (H′) in each population expected under HWE against the distance from the centroid (ri) were created in Microsoft Excel 2016.

Y-chromosome haplotype diversity was calculated as *n(1-Σpi*^2^*)/(n-1)* (where *n* is the sample size and *pi* is the frequency of the i^th^ haplotype^35^). For statistical purposes, the length of DYS389II was subtracted from that of DYS389I^36^. The YHRD database (http://www.yhrd.org) was used to search global haplotype frequency distributions. Y-Hgs were inferred using Whit Athey’s Haplogroup Predictor (http://www.hprg.com/hapest5/index.html), and Native American specific Hg was confirmed with the SNP M3-Q3. A matrix of normalized Slatkin’s genetic distances was represented as an MDS plot using XLSTAT (Addinsoft Corp) software.

MJ networks were obtained with Network 5.0.1.1 software (http://www.fluxus-engineering.com/ ^37^). Y-STR mutations were weighted according to Qamar *et al*.^38^

## Supplementary information

Dataset 1-a

Dataset 1-b

Dataset 2

Dataset 3

## Data Availability

The datasets analysed during the current study are available from the corresponding author upon reasonable request or are included in this published article (and its supplementary information files).
